# Visualized analysis of core themes and emerging frontiers in global chikungunya virus studies

**DOI:** 10.3389/fmicb.2025.1707833

**Published:** 2026-02-09

**Authors:** Jing Tian, Yonggang Li, Yuanlong Zhao, Xiaoli Tao

**Affiliations:** 1 Department of Immunity and Pathogenic Microbiology, Jinzhou Medical University, Jinzhou, China; 2 Collaborative Innovation Centre for Prevention and Control of Zoonoses, Jinzhou Medical University, Jinzhou, China; 3 School of Basic Medical Sciences, Shenyang Medical College, Shenyang, China

**Keywords:** chikungunya virus, bibliometrics, trends, VOSviewer, CiteSpace

## Abstract

**Background:**

Chikungunya virus (CHIKV) is a mosquito-borne alphavirus that has caused multiple outbreaks worldwide in recent years, resulting in millions of infections and severe complications such as chronic arthritis, posing an ongoing threat to global public health.

**Objective:**

This study employs bibliometric methods to systematically analyze the core themes, development trends, and research frontiers in CHIKV research, aiming to provide a scientific basis for research planning and prevention strategies.

**Methods:**

Based on the Web of Science Core Collection database, a total of 3,709 relevant publications from 2015 to 2024 were included. Visualization tools such as VOSviewer and CiteSpace were used to analyze countries/regions, institutions, authors, journals, keywords, and co-citation networks.

**Results:**

The results indicated that the United States, Brazil, and India were the most productive countries, while French institutions stood out in research output. Scott C. Weaver was identified as the most prolific author. Journals including PLOS Neglected Tropical Diseases, Viruses, and the Journal of Virology demonstrated significant influence in this field. Co-citation and keyword cluster analyses revealed that phylogenetic analysis, epidemiology, pathogenesis, drug therapy, and vaccine development represent current research hotspots. International collaboration plays a key role in promoting global phylogenetic studies and data integration.

**Conclusions:**

CHIKV research is expanding from epidemiology to pathogenic mechanisms, targeted therapies, and public health prevention strategies. Future efforts should focus on viral evolution mechanisms, host immune interactions, multi platform vaccine development, and the construction of global risk prediction models to address the persistent challenges posed by CHIKV outbreaks.

## 1 Introduction

Chikungunya virus (CHIKV) is a mosquito-borne alphavirus belonging to the family Togaviridae (Strauss and Strauss, 1994), named after the debilitating fever and severe arthralgia it causes (Robinson, 1955). The virus was first identified in 1952 during an outbreak in Tanzania, Africa (Lumsden, 1955). For decades, CHIKV remained endemic primarily in parts of Africa and Asia. However, in 2004, a key mutation (A226V) in the E1 gene of the virus emerged on the coast of Kenya, enhancing its adaptability to mosquito vectors and facilitating more efficient transmission (Tsetsarkin et al., 2007). This genetic change triggered an unprecedented global resurgence, spreading rapidly from Indian Ocean islands to Southeast Asia, the Americas, and some temperate regions of Europe. To date, CHIKV has been reported in over 100 countries and territories, causing approximately one million infections annually worldwide, posing a significant and ongoing public health challenge on a global scale (Cai et al., 2022).

The epidemiological situation of CHIKV is complex and continually changing. Global warming has expanded the habitat range of *Aedes* spp. vectors, such as *Aedes aegypti* and *Aedes albopictus*. These vectors possess strong ecological plasticity, exhibit opportunistic feeding behavior, and demonstrate high flexibility in utilizing both urban and natural breeding sites. These characteristics facilitate their widespread dispersal and successful adaptation across tropical, subtropical, and even temperate regions (Bierbrier et al., 2024). In addition, frequent international travel and trade have accelerated the long-distance spread of the virus, resulting in highly unpredictable outbreaks and extensive geographical distribution (Bierbrier et al., 2024). Compounding the challenge, a considerable proportion of patients progress from acute infection to chronic and disabling arthritis, with symptoms that can persist for months or even years. This leads to significant workforce loss, reduced quality of life, and imposes a substantial economic disease burden on society (Wilder-Smith and Wilder-Smith, 2024).

In terms of pathogenesis, CHIKV infection directly causes viremia and cytopathic effects, and triggers a strong host immune-inflammatory response. The virus enters the human body through the bite of an infected *Aedes* mosquito, invading the skin and relying on the E2 glycoprotein to bind host cell glycosaminoglycans (GAGs) and the MXRA8 receptor for cellular entry (Hoornweg et al., 2016; McAllister et al., 2020; Zhang et al., 2018). Viral RNA is translated into non-structural proteins (nsPs), and subgenomic RNA is further translated into structural proteins that govern viral assembly and release (Freppel et al., 2024). The inflammatory response induced by the virus is often accompanied by elevated levels of immune mediators and the infiltration of immune cells into infected joints and surrounding tissues (Burt et al., 2017). Fatal outcomes due to CHIKV are associated with increased levels of pro-inflammatory cytokines and chemokines (de Souza et al., 2024a). The specific mechanisms underlying acute and chronic arthropathic damage, particularly the relationship between viral persistence and autoimmune responses, remain major focuses and challenges in current research.

Currently, there are no FDA approved drugs for the treatment of CHIKV infection, and alternative therapeutic options against this viral disease remain limited. Research indicates that developing drugs targeting proteins encoded by the CHIKV genome, including non-structural proteins nsP1 to nsP4 and structural proteins such as E3, E2, E1, C, and 6 K, represents a promising strategy to improve treatment efficacy and reduce mortality in CHIKF (Wang et al., 2024). Targeted disruption of nsP3 assembly has been shown to significantly inhibit viral replication and transcription (Kril et al., 2024). Studies by Martins et al. (2020) have identified natural compounds such as chloroquine, apigenin, and chrysin as exhibiting antiviral activity against CHIKV (Kril et al., 2024). During the viremic phase, the use of anti-CHIKV monoclonal antibodies has successfully reduced the spread of the virus to distal joints and tissues (Fox et al., 2019; Broeckel et al., 2017). The long non-coding RNA ALPHA (prohibiting human alphaviruses) interacts with the CHIKV genomic RNA and specifically targets viral RNA replication (Basavappa et al., 2022). Although researchers have developed various vaccine candidates, including live attenuated vaccines, virus-like particle vaccines, and mRNA vaccines, which show promising immunogenicity (Ng and Renia, 2024; Maure et al., 2024; Schneider et al., 2023), the unpredictable geographic spread of outbreaks and the genetic diversity of the virus pose major challenges for Phase III efficacy trials and broad applicability. Numerous questions remain regarding vaccine safety and immunogenicity in children, pregnant women, and immunocompromised populations, as well as how vaccine coverage may alter population level immunity and transmission patterns of alphaviruses (Weber et al., 2024).

This study aims to systematically sort out the molecular biological features and epidemiological evolution trends of CHIKV using bibliometrics, conduct an in-depth exploration of its immunological and virological mechanisms of pathogenesis, and comprehensively review the latest progress and existing obstacles in the field of current antiviral drug and vaccine research and development. By synthesizing current scientific evidence, we seek to offer valuable insights to guide future basic research on CHIKV and inform the design of therapeutic and preventive strategies, ultimately contributing to the effective control of this significant emerging infectious disease.

## 2 Methods

### 2.1 Data sources and search strategy

**Figure 1:**
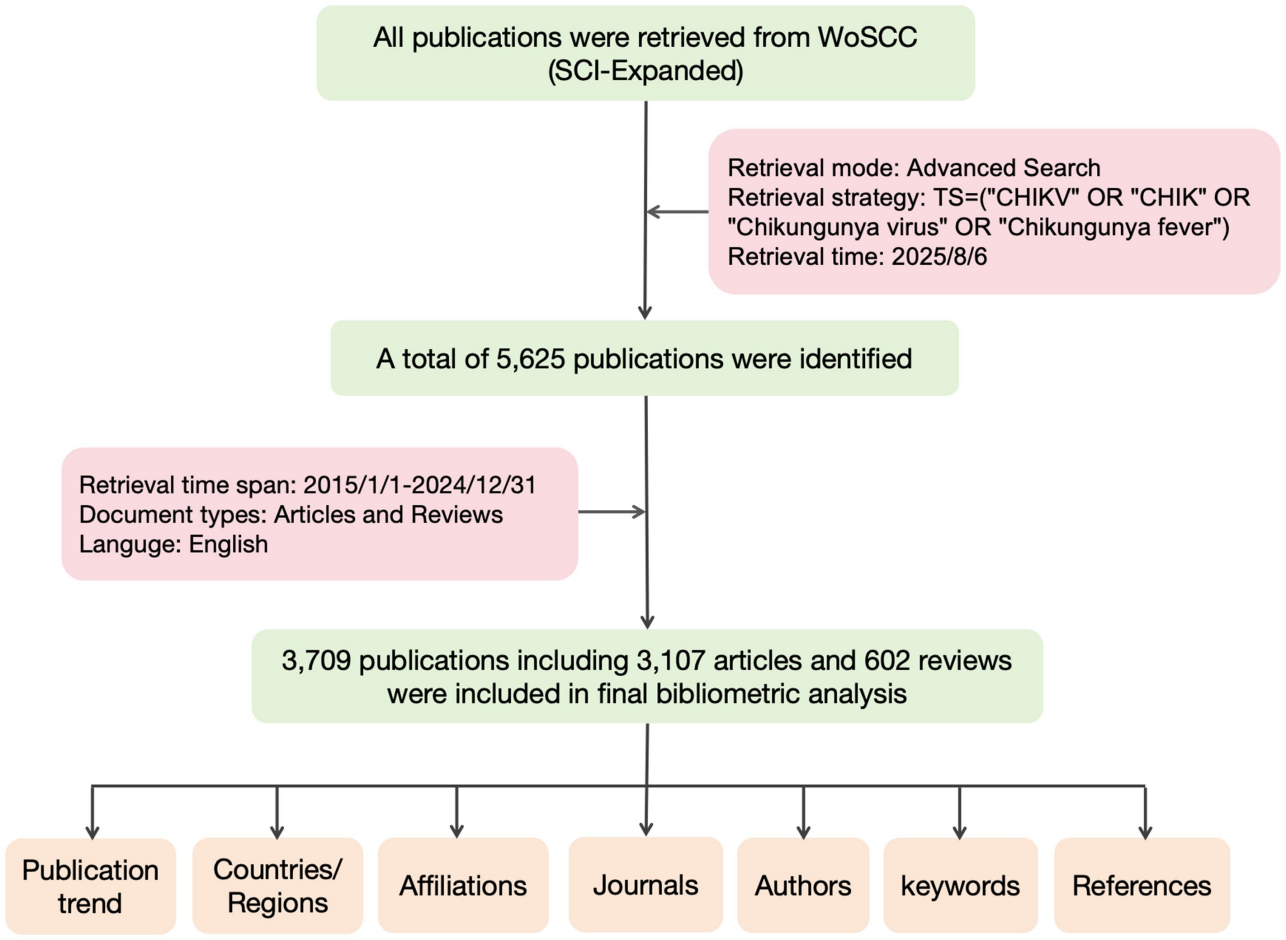
Flowchart of inclusion and exclusion criteria.

The literature search for this study was conducted using the Web of Science Core Collection (WoSCC) database. This database was selected as the sole source for three primary reasons: First, its rigorous journal selection process ensures the inclusion of high-quality, influential literature, which forms a reliable core dataset for analysis. Second, WoS provides standardized and complete citation data, which is crucial for the accuracy of co-citation and collaboration network analyses. Third, using WoS ensures methodological consistency and comparability with the vast majority of existing bibliometric studies. The retrieval was performed on August 6, 2025, using the Topic (TS) field with the following query: TS = (‘CHIKV’ OR ‘Chikungunya virus’ OR ‘Chikungunya fever’ OR ‘CHIK’). Articles published between January 1, 2015, and December 31, 2024, were included, yielding an initial result of 5,625 publications. A 10-year time span was selected to ensure both the timeliness of research trends and the representativeness of the data. This duration is widely recognized in bibliometric studies as suitable for capturing emerging research directions while maintaining consistency and analytical depth. To ensure data integrity and reproducibility, the search was completed within a single day, and only English-language original research articles and reviews were considered. Two researchers independently screened the literature. Records unrelated to CHIKV research were excluded, along with non-qualifying publication types such as conference abstracts, case reports, letters, and preprints. A total of 3,709 eligible publications were ultimately included. The detailed screening process is illustrated in Figure 1.

### 2.2 Bibliometric and visualization analysis

To examine the development, evolutionary trends, and emerging research fronts in CHIKV studies over the past decade, this study employed VOSviewer (version 1.6.20), CiteSpace (version 6.4.R1), and the online platform https://bibliometric.com/application. The analysis covered multiple dimensions, including institutional and journal distributions, country/region contributions, author collaborations, keyword clustering, and characteristics of highly cited references.

CiteSpace (version 6.4.R1) was used to visualize the analysis of scientific literature, citation counts, total publications, key disciplines and journals, research institutions and collaborations, as well as author analyses. The software also supports keyword frequency analysis, keyword clustering, and burst detection. The time span (2015–2024) was divided into 1-year slices. For each slice, the top 50 cited references or occurring keywords were selected. The resulting networks were pruned using the Pathfinder algorithm. In co-occurrence maps generated by CiteSpace, node size corresponds to the number of publications, connections between nodes represent collaborations, and line thickness reflects the frequency of cooperation—more connections and thicker lines indicate stronger co-citation or co-occurrence relationships. Furthermore, the knowledge structure of a field can be visualized through cited references, where highly cited works are regarded as classic and authoritative. In the citation network, node size is proportional to co-citation frequency, and links between nodes indicate co-citation relationships. Overall, CiteSpace provides an intuitive representation of core authors and keyword networks within a specific research domain.

VOSviewer (version 1.6.20) was also extensively applied in the analysis and visualization of scientific literature, keywords, author relationships, as well as conducting co-occurrence analysis, network visualization, heat maps, and cluster analysis. VOSviewer was configured with the following thresholds: a minimum occurrence of 47 for keyword co-occurrence analysis, a minimum citation count of 90 for reference co-citation analysis, and inclusion of the top 30 countries for international collaboration mapping. In its co-citation maps, nodes represent individual units (e.g., publications, keywords, or authors), and nodes of the same color belong to a related cluster. Node diameter corresponds to the number of publications or citations, with larger nodes indicating key research themes. The distance between nodes reflects the strength of their relationship—shorter distances imply stronger associations. In this study, VOSviewer was used to generate country/agency distribution maps, international collaboration networks, and visualizations of keywords and co-cited references. Additionally, Microsoft Office Excel 2021 was employed for organizing and statistically describing publication-related metrics.

## 3 Results

### 3.1 Overview of publications on CHIKV

Based on the keyword search, a total of 5,625 publications were initially identified. Following screening by publication year, document type, and language, 3,709 research articles and reviews published in the last decade (2015–2024) were ultimately selected from the Science Citation Index Expanded (SCI-Expanded) edition of the Web of Science Core Collection. These publications have accumulated 13,028 total citations, with an average of 3.51 citations per article. The H-index for the entire dataset was 29. The detailed screening process is presented in Table 1.

**Table 1:** Flowchart of the screening process.

Set	Results	Refinement
1	5,625	TOPIC: (TS = “CHIKV” OR “Chikungunya virus” OR “Chikungunya fever” OR “CHIK”) Indexes = SCI-EXPANDED
2	4,153	Refined by PUBLICATION YEARS: (From 2015 to 2024)
3	3,754	Refined by DOCUMENT TYPES: (ARTICLES OR REVIEW ARTICLES)
4	3,709	Refined by LANGUAGES: (ENGLISH)

### 3.2 Annual publication trends

**Figure 2:**
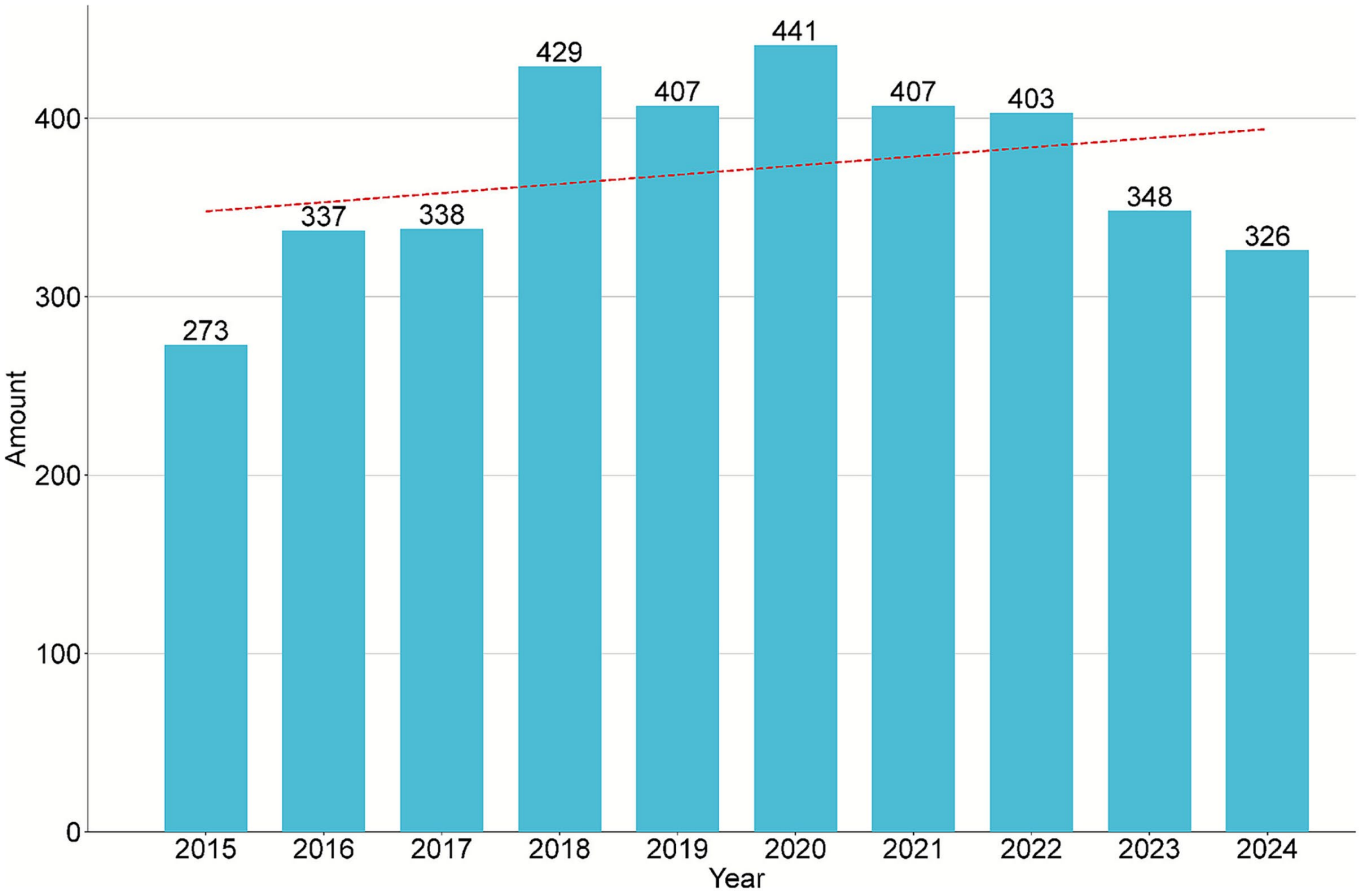
Annual distribution and growth trend of publications from 2015 to 2024.

The annual number of publications increased from 273 in 2015 to 326 in 2024, demonstrating a modest overall upward trend over the past decade. The number of CHIKV related publications peaked at 441 in 2020 (Figure 2). This irregular trend may be indirectly attributed to the COVID-19 pandemic, which stimulated research output in areas such as COVID-19 and CHIKV co-infections, differences in clinical manifestations, diagnostic approaches, and potential immune interference.

### 3.3 Contributions of countries/regions to the global publication

**Figure 3:**
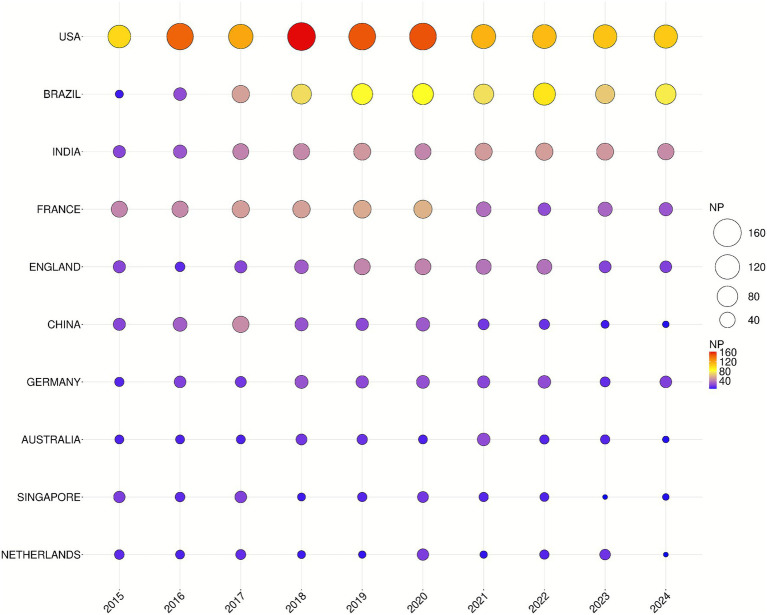
Top 10 countries in terms of annual publications on CHIKV research from 2015 to 2024. The circle’s size and colors show the number of papers. The larger the circle, the color from blue to red, the higher the NP issued in that country.

Analysis at the country/region level indicates that the 3,709 publications originated from 163 countries or regions. Among the top 10 countries/regions in terms of annual publication output in CHIKV research between 2015 and 2024, the U.S. led with the highest number of publications, totaling 1,266 (34.13%), followed by Brazil with 641 (17.28%) and India with 432 (11.65%). Publications from the U.S. accumulated 35,464 citations, accounting for 32.93% of the total citations. Those from Brazil received 15,891 citations (14.76%), while Indian publications were cited 6,794 times (6.31%). The U.S. also achieved the highest H-index of 86, significantly exceeding that of other countries/regions. Singapore recorded the highest average citations per article at 50.88, although its total publication output and H-index were relatively lower compared to the U.S. (Figure 3 and Table 2).

**Table 2:** Top 10 countries/regions with the highest productivity.

Rank	Country/Region	NP	NC	H-index	Average citation per item
1	USA	1,266	35,464	86	32.33
2	BRAZIL	641	15,891	53	28.13
3	INDIA	432	6,794	41	17.18
4	FRANCE	431	15,293	60	37.65
5	ENGLAND	295	11,814	51	41.78
6	CHINA	232	5,826	26	38
7	GERMANY	213	6,385	41	31.05
8	AUSTRALIA	151	3,526	34	24.99
9	SINGAPORE	136	6,701	41	50.88
10	NETHERLANDS	126	3.687	31	30.36

**Figure 4:**
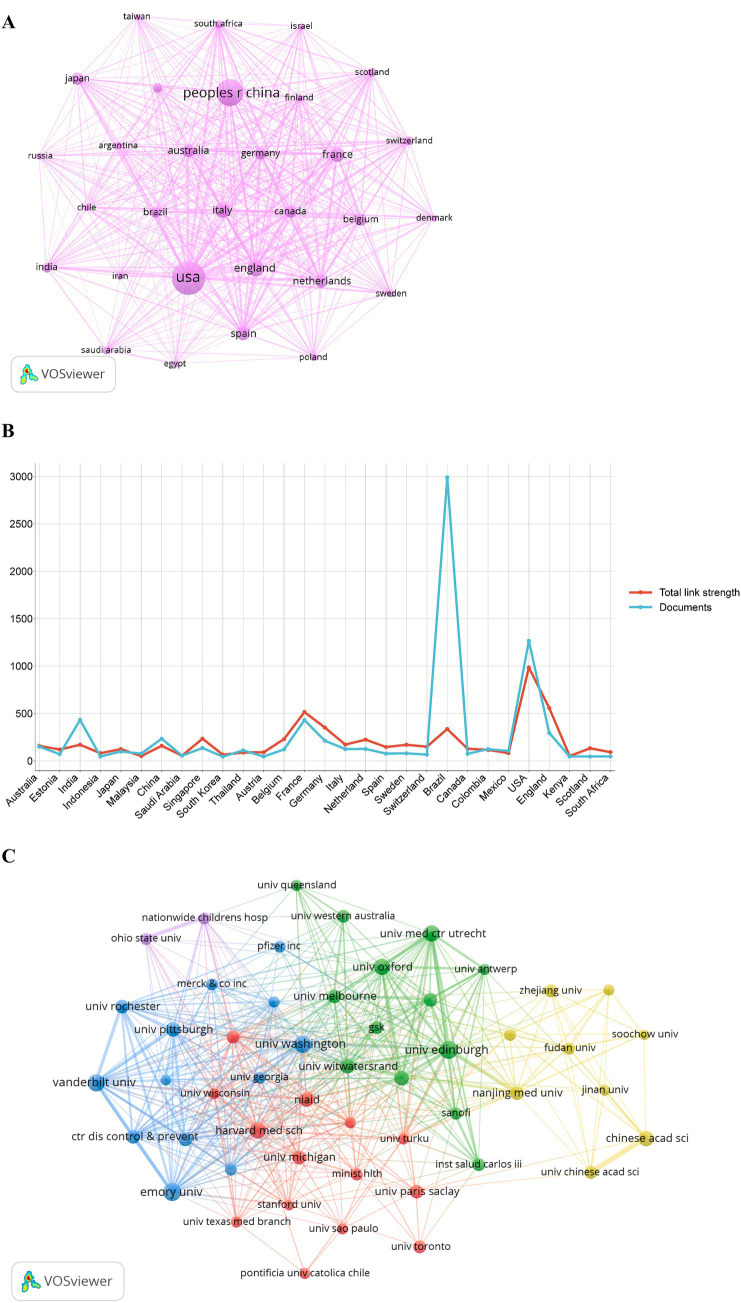
Collaborative network of countries/regions and affiliations. **(A)** Collaboration network of the top 30 most productive countries/regions. **(B)** Total link strength and collaborative link count (“Documents” metric from VOSviewer) of the top 30 most productive countries/regions. **(C)** Collaboration network of the top 50 affiliations.

Efficient collaboration among institutions and countries is crucial for fostering academic exchange and advancing scientific research. Figure 4A presents the collaboration network among the top 30 countries/regions with the highest publication output in the field of Chikungunya virus from 2015 to 2024. The U.S. and China emerged as the two leading contributors, with collaboration intensity substantially surpassing that of other nations. Additionally, the U.S. maintained strong collaborative ties with Brazil, Sweden, Colombia, China, and South Korea, while Australia engaged in extensive partnerships with the U.S., the U.K., France, Brazil, India, and China, among others.

Figure 4B illustrates the total link strength and the number of collaborative links (“Documents” metric) for the top 30 most productive countries, revealing distinct collaboration profiles. The U.S. demonstrates the highest total link strength (985), far exceeding that of Brazil (336) and India (170), underscoring its central and highly integrated role in the global research network. Conversely, Brazil leads in the number of collaborative links (2,990) but exhibits a comparatively moderate total link strength (336). This discrepancy suggests that Brazil’s numerous international collaborations may translate into less cohesive integration within the broader network compared to the U.S.

### 3.4 Institutional analysis

Among the top 10 institutions with the highest number of publications (NPs) in the field of CHIKV research, Fundação Oswaldo Cruz from Brazil ranked first with 237 publications, followed by Université Paris Cité (191 publications) and the Pasteur Network (184 publications) from France (Table 3). Excluding the Pasteur Network, French institutions accounted for five of the top 10 positions, while the U.S. and Brazil each contributed two institutions, highlighting the strong research capacity and high academic standard of French institutions in Chikungunya virus studies.

**Table 3:** Top 10 most productive affiliations.

Rank	Affiliations	Country/Region	NP	NC	H-index	Average citation per item
1	Fundação Oswaldo Cruz	Brazil	237	8,278	39	37.02
2	Université Paris Cité	France	191	7,989	47	43.3
3	Pasteur Network	/	184	7,285	45	41.01
4	Institut national de la santé et de la recherche médicale (Inserm)	France	177	4,830	39	28.69
5	Institut Pasteur PARIS	France	148	6,330	41	44.12
6	Centre national de la recherche scientifique (CNRS)	France	144	5,622	38	40.27
7	University of Texas System	USA	137	5,855	44	45
8	Institut de Recherche pour le Développement (IRD)	France	126	3,714	30	30.39
9	University of Texas Medical Branch	USA	126	5,515	44	46.17
10	University of São Paulo	Brazil	119	3,564	26	30.95

Furthermore, we analyzed collaborative relationships among these research institutions (Figure 4C). The results revealed that Institut Pasteur maintains close collaborations with Aix-Marseille University, University of Oxford, Emory University, Mahidol University, and Université de Montpellier. Similarly, Universidade Federal de Minas Gerais demonstrated strong collaborative ties with Universidade de São Paulo, Fundação Oswaldo Cruz, Universidade Federal da Bahia, Universidade Federal do Rio de Janeiro, Universidade Federal de Pernambuco, and Yale University.

### 3.5 Author analysis

The top 10 most prolific authors in the field of CHIKV research contributed a total of 468 publications, accounting for 12.6% of the total output over the past decade. Scott C Weaver from the University of Texas Medical Branch at Galveston ranked first with 75 publications, followed by Andres Merits from the University of Tartu (71 publications) and Lisa F.P. Ng from the Agency for Science, Technology and Research (54 publications). Notably, Scott C Weaver also led in total citations (NC = 3,657) and H-index (Weaver et al., 2018), while ranking second in average citations per article (51.12), reflecting the broad recognition of his research among peers (Table 4). Furthermore, these top 10 authors are affiliated with institutions across seven different countries/regions, indicating a globally distributed research effort dedicated to the study of CHIKV.

**Table 4:** Top 10 authors with the most publications.

Rank	Author	NP	NC	Country/Region	Affiliation	H-index	Average citation per item
1	Scott C Weaver	75	3,657	USA	University of Texas Medical Branch Galveston	36	51.12
2	Merits, Andres	71	2,609	Estonia	University of Tartu	33	40.49
3	Ng, Lisa F.P.	54	1,971	Singapore	Agency for Science Technology Research (A*STAR)	25	38.56
4	Failoux, Anna-Bella	50	1,799	France	University Paris Cite	22	37.64
5	Michael Diamond	43	2,406	USA	Washington University Wustl	30	59.6
6	Neyts, Johan	41	1,159	Netherlands	Ku Leuven	21	30.2
7	Xavier de Lamballerie	41	1,153	France	Aix Marseille Université	19	28.71
8	Delang, Leen	33	741	Netherlands	Ku Leuven	19	24.7
9	Leyssen, Pieter	30	1,111	Netherlands	Ku Leuven	19	38.47
10	Mahalingam, Suresh	30	1,031	Australia	Griffith University	16	36.47

### 3.6 Journal analysis

Among the top 10 journals that published the most CHIKV research over the past decade, *PLOS Neglected Tropical Diseases* ranked first with 258 publications (2024 Impact Factor: IF 3.4), followed by *Viruses* (Basel) with 220 articles (IF 3.5) and the *Journal of Virology* with 111 articles (IF 3.8) (Table 5). *PLOS Neglected Tropical Diseases* specializes in publishing groundbreaking research in the field of neglected tropical diseases. Its dedicated section, “Virus,” focuses on studies related to arboviruses, zoonotic viruses, and other emerging and re-emerging viral infectious diseases, with particular emphasis on the transmission mechanisms, control strategies, and innovative therapies for viruses such as dengue and chikungunya in resource-limited settings. Among the top 10 journals, the majority had an Impact Factor higher than 3.000. Approximately 29.60% of CHIKV related publications (1,098 out of 3,709) were published in these top 10 journals.

**Table 5:** Top 10 most-published journals.

Rank	Journal	NP	NC	IF (2024)	H-index	Average citation per item
1	PLOS NEGLECTED TROPICAL DISEASES	258	8,392	3.4	48	33.68
2	VIRUSES BASEL	220	3,430	3.5	30	16.52
3	JOURNAL OF VIROLOGY	111	3,141	3.8	37	30.09
4	PLOS ONE	107	2,193	2.6	30	20.86
5	AMERICAN JOURNAL OF TROPICAL MEDICINE AND HYGIENE	97	1,598	1.6	24	16.85
6	SCIENTIFIC REPORTS	87	2,391	3.9	30	27.82
7	PARASITES VECTORS	66	1,427	3.5	23	22.06
8	ANTIVIRAL RESEARCH	57	2,307	4	28	41.56
9	ACTA TROPICA	48	989	2.5	19	20.92
10	PATHOGENS	47	528	3.3	13	11.4

### 3.7 Analysis of global citation scores (GCS) of publications

**Figure 5:**
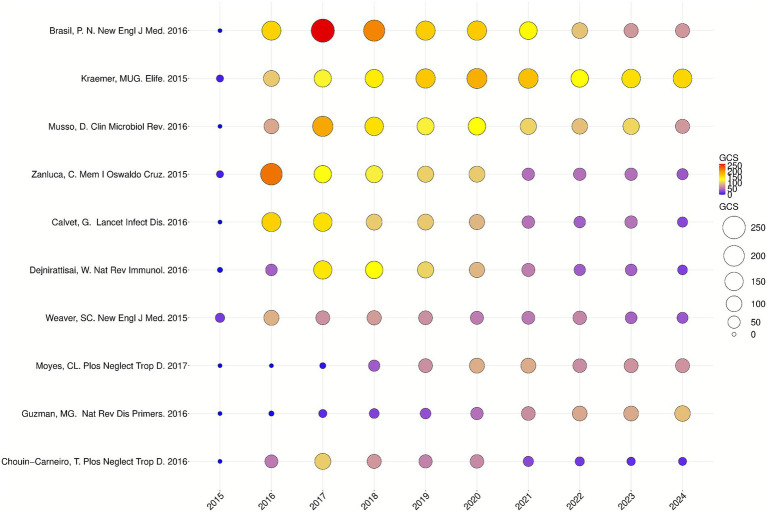
Top 10 articles with the highest GCS by year. The size and color of the circles represent the articles’ GCS. Larger circles and the color gradient from blue to red indicate higher GCS values and greater influence within the research field.

The citation count of individual articles reflects hotspots and trends in CHIKV research. Table 6 lists the top 10 most cited papers in this field, and Figure 5 illustrates the annual variation in GCS for these 10 highly influential publications. The most cited article (1,353 citations) was published by Kraemer, Moritz U.G. et al. in 2016 in *New England Journal of Medicine* (IF 78.5). The team conducted a follow-up study in Rio de Janeiro on Zika virus infection in pregnant women, systematically described maternal clinical symptoms and the impact of acute Zika virus infection on infants (Brasil et al., 2016). The second article (1,352 citations) was published by Kraemer, M.U.G. et al. in 2015 in *eLife*, systematically assessed the global distribution of *Aedes aegypti* and *Aedes albopictus*, the primary vectors for arboviruses such as dengue and chikungunya. By integrating the largest contemporary database for both species and analyzing relevant environmental variables, the study predicted their global distribution and provided a basis for defining the current spatial boundaries of local transmission of dengue and chikungunya viruses (Kraemer et al., 2015). Ranking sixth was a study by Dejnirattisai, W. et al. published in *Nature Reviews Immunology* in 2016. The study found that plasma from individuals immune to dengue virus (DENV) exhibited cross reactivity with ZIKV and could mediate antibody-dependent enhancement of ZIKV infection, providing basis for understanding pathogenesis and informing future vaccine for both ZIKV and DENV (Dejnirattisai et al., 2016). In addition, other highly cited articles focused on Zika virus detection, sequencing, and phylogenetic evolution (Musso and Gubler, 2016; Zanluca et al., 2015; Calvet et al., 2016), overviews of the epidemiology, diagnosis, and treatment of CHIKV and dengue viruses (Guzman et al., 2016; Weaver and Lecuit, 2015) and the susceptibility of *Aedes aegypti* and *Aedes albopictus* to viruses and their resistance to insecticides (Chouin-Carneiro et al., 2016; Moyes et al., 2017).

**Table 6:** The most-cited paper from 2005 to 2024.

Rank	Year	Article	IF (2024)	Total citation	Type of study
1	2016	Kraemer, Moritz U. G. et al. Zika Virus Infection in Pregnant Women in Rio de Janeiro*. New Engl J Med.* DOI: 10.1056/NEJMoa1602412	78.5	1,353	Article
2	2015	Kraemer, MUG. et al. The global distribution of the arbovirus vectors *Aedes aegypti* and Ae. albopictus. *Elife.* DOI: 10.7554/eLife.08347	–	1,352	Article
3	2016	Musso, D. et al. Zika Virus. *Clin Microbiol Rev.* DOI: 10.1128/CMR.00072-15	19.3	1,070	Review
4	2015	Zanluca, C. First report of autochthonous transmission of Zika virus in Brazil. *Mem I Oswaldo Cruz.* DOI: 10.1590/0074-02760150192	2.5	880	Article
5	2016	Calvet, G. et al. Detection and sequencing of Zika virus from amniotic fluid of fetuses with microcephaly in Brazil: a case study. *Lancet Infect Dis.* DOI: 10.1016/S1473-3099(16)00095-5	31	782	Article
6	2016	Dejnirattisai, W. et al. Dengue virus sero-cross-reactivity drives antibody-dependent enhancement of infection with zika virus. *Nat Rev Immunol.* DOI: 10.1038/ni.3515	27.6	684	Article
7	2015	Weaver, SC. et al. Chikungunya Virus and the Global Spread of a Mosquito-Borne Disease. *New Engl J Med.* DOI: 10.1056/NEJMra1406035	78.5	580	Review
8	2017	Moyes, CL. et al. Contemporary status of insecticide resistance in the major Aedes vectors of arboviruses infecting humans. *Plos Neglect Trop D.* DOI: 10.1371/journal.pntd.0005625	3.4	491	Review
9	2016	Guzman, MG. et al. Dengue infection. *Nat Rev Dis Primers.* DOI: 10.1038/nrdp.2016.55	60.6	451	Article
10	2016	Chouin-Carneiro, T. et al. Differential Susceptibilities of *Aedes aegypti* and *Aedes albopictus* from the Americas to Zika Virus. *Plos Neglect Trop D*. DOI: 10.1371/journal.pntd.0004543	3.4	435	Article

**Figure 6:**
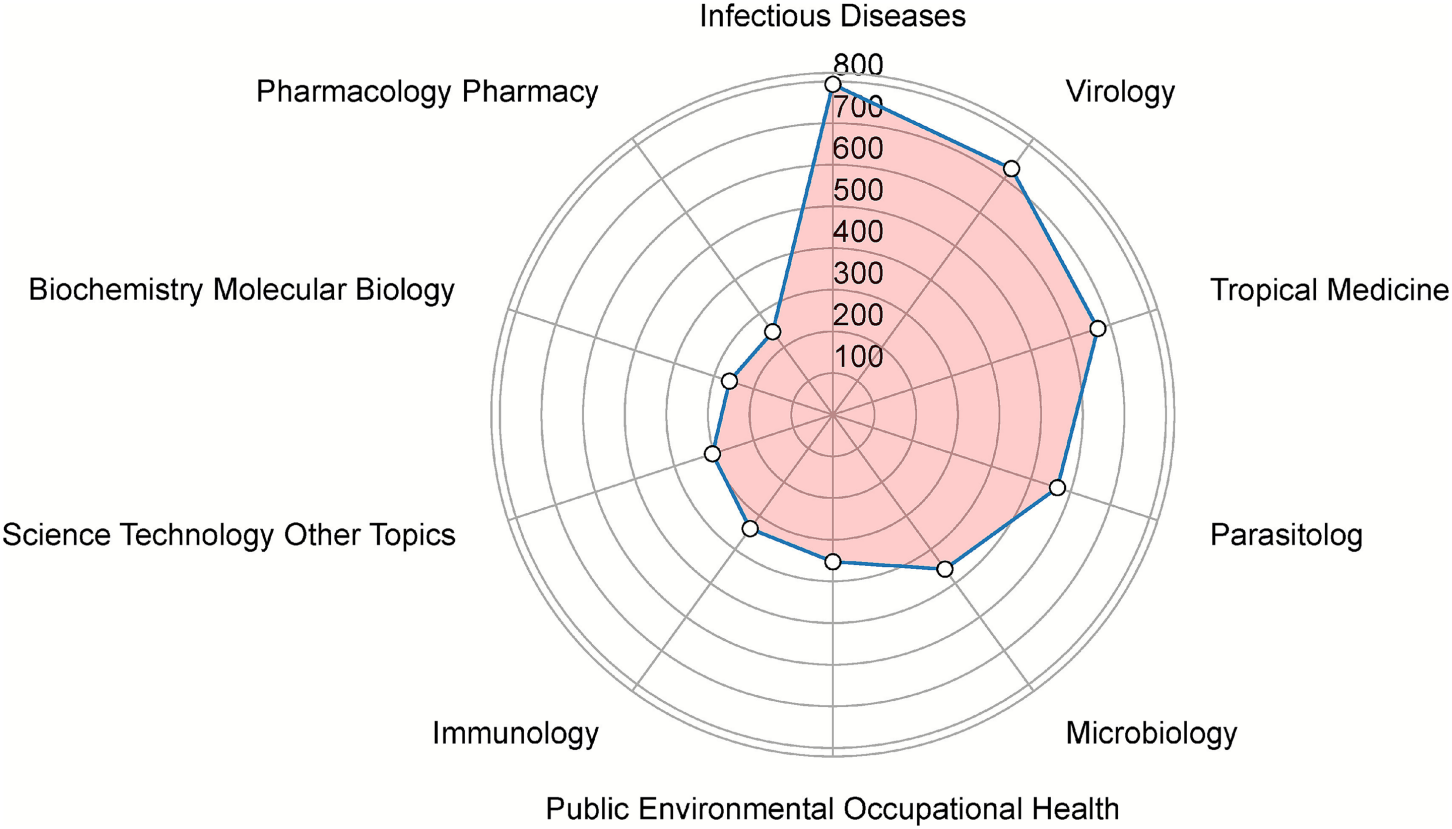
Radar map of the top 10 research productive categories on CHIKV.

Among the top 10 most productive research areas related to Chikungunya virus, the categories with the highest number of publications were: Infectious Diseases (793 articles), Virology (730 articles), Tropical Medicine (669 articles), Parasitology (567 articles), Microbiology (458 articles), Public Environmental Occupational Health (353 articles), Immunology (338 articles), Science Technology Other Topics (304 articles), Biochemistry Molecular Biology (261 articles) and Pharmacology Pharmacy (246 articles). Among all research categories, ‘Infectious Diseases’ accounted for the highest proportion, while ‘Pharmacology Pharmacy’ had the lowest proportion (Figure 6).

### 3.8 Analysis of co-cited references

**Figure 7:**
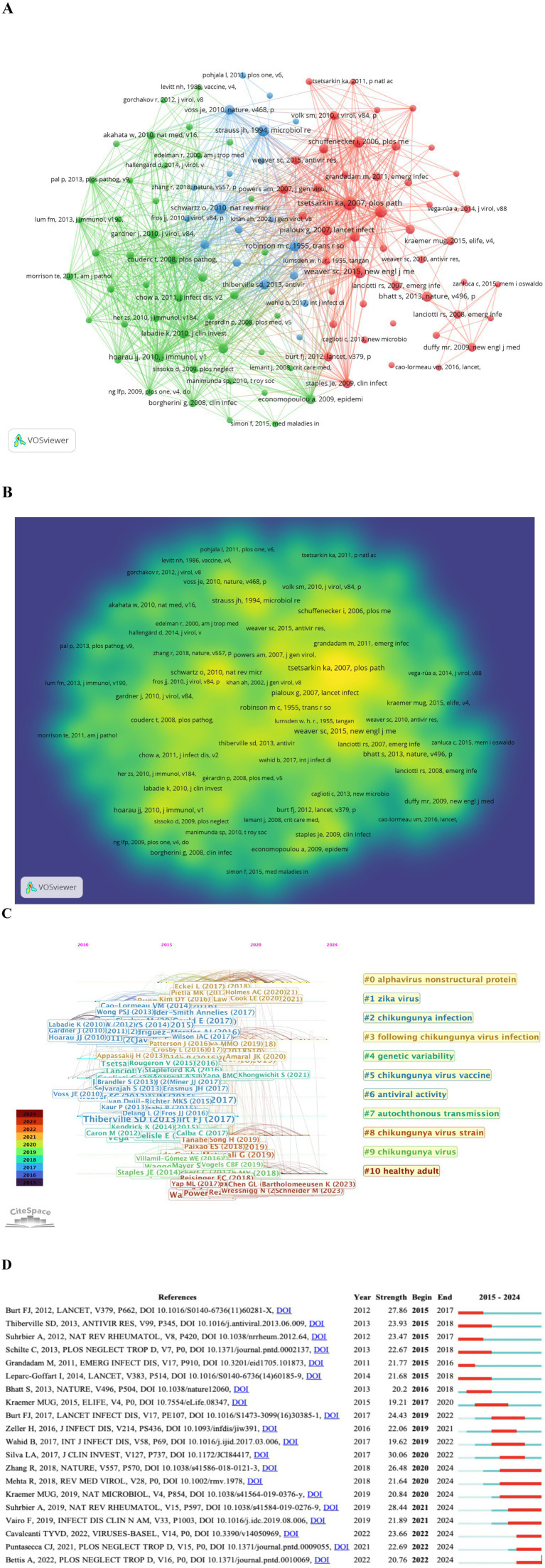
Mapping analysis based on co-cited references in CHIKV research. **(A)** Network diagram of cocited references. Cluster 1 is in red, cluster 2 is in green, and cluster 3 is in purple. **(B)** Density visualization for 110 co-cited references network map. Each keyword in the density visualization has colors that indicates its density. Yellow means appearing more frequently, while green means appearing less frequently. **(C)** Top 20 co-cited references with the strongest citation bursts. The years between “Begin” and “End” represent the period when the reference was more influential. **(D)** Top 20 references with the strongest citation bursts. Years in light green mean that the reference has not yet published, and years in dark green mean that the reference has a less influential. Instead, years in red represents that the reference has a higher influence.

Unlike global citation analysis, co-citation networks emphasize research topics closely related to a specific field. Due to the large volume of cited literature, the minimum citation threshold (NC) was set to 90. Out of 96,142 cited references, 110 were ultimately selected for co-citation analysis and divided into three clusters (Figure 7A). These 110 references are categorized by color into the following clusters. Cluster 1 (Red, 46 references) focuses on epidemiological surveillance and national disease burden of Chikungunya virus. Cluster 2 (Green, 45 references) centers on disease characteristics and complications caused by Chikungunya virus. Cluster 3 (Purple, 19 references) primarily investigates the pathogenesis and immune response mechanisms induced by Chikungunya virus.

To further explore the co-citation relationships among CHIKV-related literature, a density visualization was performed on the 110 references (Figure 7B). Density visualization helps reveal the overall research structure and highlights important research domains. Figure 7C presents the most representative references in terms of burst duration, strength, and timing. The hot topics identified through co-citation clustering include: ‘alphavirus nonstructural protein’, ‘zika virus’, ‘chikungunya infection’, ‘following chikungunya virus infection’, ‘genetic variability,” ‘chikungunya virus vaccine’, ‘antiviral activity’, ‘autochthonous transmission’, ‘chikungunya virus strain’, ‘chikungunya virus’ and ‘healthy adult’. Among these, ‘chikungunya virus strain’ and ‘healthy adult’ marked in brown, represent the most current and prominent research directions.

Figure 7D displays the top 20 references with the strongest citation bursts. The article by Silva LA et al., published in *J Clin Invest* in 2017, had the highest burst strength (30.06) during 2015–2024. This review systematically describes the epidemiology, replication cycle, pathogenesis, and host immune response of CHIKV, discusses prospects for effective vaccines, and highlights key questions for future research (Silva and Dermody, 2017). The second strongest burst was observed for the article by Suhrbier A et al., published in *Nat Rev Rheumatol* in 2019, which elaborates on the changing global distribution of mosquito vectors such as *Aedes aegypti* and *Aedes albopictus* and future transmission trends of arboviral diseases including chikungunya and Zika virus (Suhrbier, 2019). The study by Cavalcanti TYVD et al., published in *Viruses* in 2022, describes the epidemiology, pathogenesis, and current vaccine development for chikungunya virus (de Lima Cavalcanti et al., 2022). These highly burst strength publications provide in-depth insights into CHIKV from multiple perspectives, including epidemiology, vector distribution, pathogenesis, drug therapy, and vaccine development.

### 3.9 Research hotspot analysis

**Figure 8:**
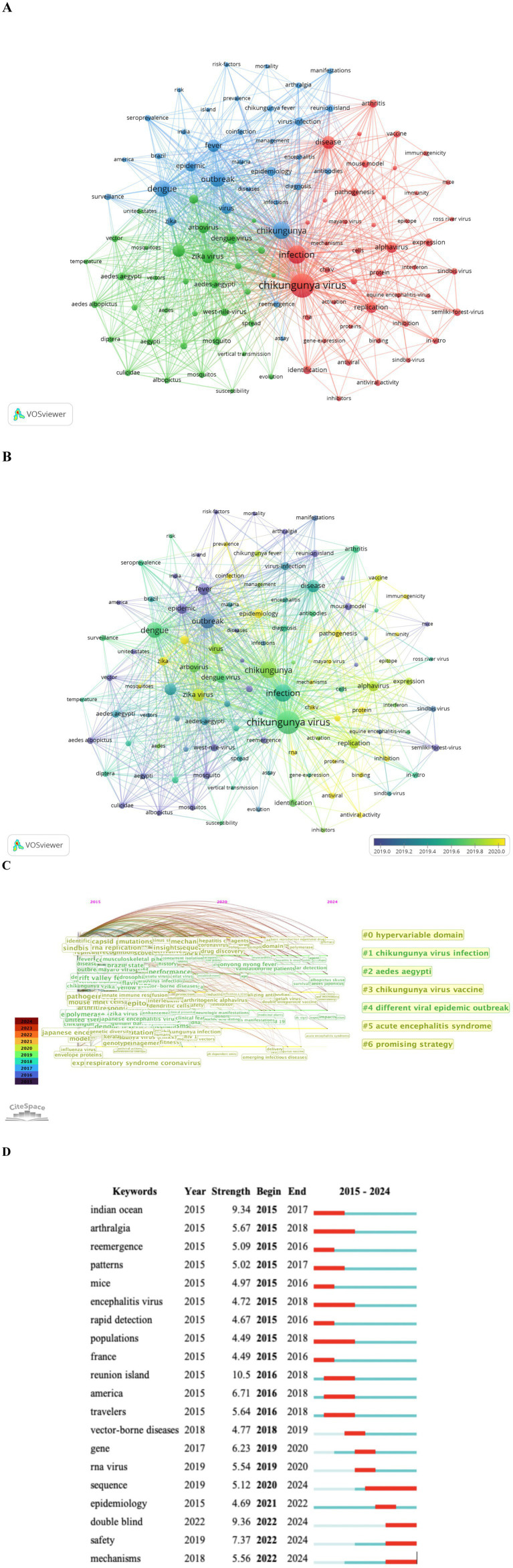
Keyword mapping of CHIKV research. **(A)** Network diagram of keywords. Cluster 1 is in red, cluster 2 is in green, and cluster 3 is in blue. The size of the nodes indicates occurrence frequency. **(B)** Keywords visualization according to the APY. The different colors indicate the relevant year of publication. Yellow keywords came later than purple keywords. **(C)** Timeline distribution of keyword cluster analysis. **(D)** Top 20 keywords with the strongest citation bursts. The years between “Begin” and “End” represent the period when the keyword was more influential. Years in light green mean the keyword has not yet appeared, years in dark green mean the keyword has a less influence, and years in red represents more influential keywords.

The co-occurrence analysis of keywords extracted from the titles and abstracts of 10,158 publications. A total of 111 keywords that appeared more than 47 times were identified and grouped into three clusters (Figure 8A). Cluster 1 (Red, 44 items) focuses on the pathogenic mechanisms and complications of CHIKV. Cluster 2 (Green, 34 items) emphasizes the transmission routes and vector distribution of CHIKV. Cluster 3 (Blue, 33 items) concentrates on epidemiological surveillance and outbreak-related studies of CHIKV. Figure 8B color-codes all keywords by their average publication year (APY) to indicate recency. Apart from ‘epidemiology’ (Cluster 3, APY: 2020.19), which appeared 211 times, the most recent keywords include: ‘arboviruses’ (Cluster 2, APY: 2020.12, 197 occurrences), ‘pathogenesis’ (Cluster 1, APY: 2020.10, 157 occurrences), ‘antiviral activity’ (Cluster 1, APY: 2020.14, 88 occurrences), ‘coinfection’ (Cluster 3, APY: 2020.17, 84 occurrences), ‘immunogenicity’(Cluster 1, APY: 2020.66, 67 occurrences), ‘prevalence’ (Cluster 3, APY: 2020.15, 60 occurrences).

Furthermore, Figure 8C indicates that keywords such as ‘hypervariable domain’, ‘chikungunya virus infection’, ‘*aedes aegypti*’, ‘chikungunya virus vaccine’, ‘different viral epidemic outbreak’, ‘acute encephalitis syndrome’ and ‘promising strategy’ have consistently attracted significant attention in CHIKV research. Among the top 20 keywords with the strongest citation bursts (Figure 8D), “indian ocean,” ‘arthralgia’ and ‘reemergence’ ranked highest in burst strength, while ‘sequence’, ‘double blind’, ‘safety’ and ‘mechanisms’ emerged as new hotspot keywords within the past year.

Synthesizing the results from Figures 8A–D, it is evident that phylogenetic analysis, pathogenic mechanisms, drug therapy, and vaccine development remain core themes of ongoing interest in CHIKV research.

## 4 Discussion

This study analyzed 3,709 publications through bibliometric mapping and visualization, employing quantitative, qualitative, and integrative research methods to systematically evaluate the research progress, hotspots, and future directions of CHIKV. To our knowledge, this is the first bibliometric study conducted in this research field.

The annual number of publications showed a gradually stabilizing growth pattern, increasing from 273 in 2015 to 326 in 2024, reflecting a modest upward trend over the past decade. This indicates sustained academic interest in CHIKV research and a gradual expansion of research scale. The U.S. ranked first in publication output with 1,266 articles (34.13%), largely attributable to the high productivity of the University of Texas System (137 articles) and the University of Texas Medical Branch (126 articles) in this field. Brazil ranked second with 641 articles (17.28%), followed by India with 432 articles (11.65%). Among the top 10 institutions, French institutions accounted for five, excluding the Pasteur Network, while the U.S. and Brazil each contributed two. Notably, India, despite ranking third in total publication output, had no institutions in the top 10. This suggests that although India has a high overall output, research achievements may be dispersed across numerous universities and research institutes, lacking internationally competitive core flagship institutions. This dispersion may prevent high-quality research from being concentrated in a few leading centers, thereby affecting institutional rankings. Institutions in France and the U.S. generally possess strong international collaboration networks and resource integration capabilities. In contrast, Indian institutions may have relatively weaker participation in international collaborations, joint research projects, or global scientific networks, limiting their international visibility and influence. India’s high number of collaborative documents relative to its total link strength indicates a tendency among Indian research institutions to establish deep and stable partnerships with a limited number of core collaborators, reflecting a relatively limited diversity in its international collaboration network. The U.S. maintained close research exchanges with Brazil, Sweden, Colombia, China, and South Korea, while Australia frequently collaborated with the U.S., the U.K., France, Brazil, India, and China, among others. In terms of institutional collaborations, the Institut Pasteur maintained close relationships with Aix-Marseille University, the University of Oxford, Emory University, Mahidol University, and the Université de Montpellier. Similarly, the Universidade Federal de Minas Gerais collaborated extensively with the Universidade de São Paulo, Fundação Oswaldo Cruz, the Universidade Federal da Bahia, the Universidade Federal do Rio de Janeiro, the Universidade Federal de Pernambuco, and Yale University. Professor Scott C. Weaver published the highest number of related articles (75 papers). His research primarily focuses on the evolution and epidemiology of arboviruses (Weaver and Lecuit, 2015; Weaver et al., 2018; Weaver and Forrester, 2015; de Souza et al., 2024b), pathophysiology and factors contributing to severe CHIKV infection (de Souza et al., 2024a), mechanisms of mosquito infection and transmission (Tsetsarkin et al., 2016; Weaver et al., 2020; Chen et al., 2021), clinical features and complications (Langsjoen et al., 2016; Cabie et al., 2015), treatment strategies (Jin et al., 2018; Langsjoen et al., 2017), as well as vaccine development and efficacy evaluation (Erasmus et al., 2016; Rossi et al., 2019; Erasmus et al., 2017).

The findings from our bibliometric analysis of countries, institutions, authors, and journals provide valuable insights for designing innovative collaboration models that can enhance cooperative efficiency and drive future advancements in CHIKV research. At the country level, the dominant roles of the U.S., Brazil, and India in publication output, coupled with their distinct collaboration patterns, suggest the need for strategic partnership frameworks. For instance, while the U.S. exhibits broad and intensive international linkages, Brazil and India show deeper but narrower collaborative ties. This indicates an opportunity for hub-and-spoke collaboration models, where highly connected countries (e.g., U.S., France) serve as hubs to facilitate multi-directional knowledge exchange and resource sharing with regionally focused partners. Such models could help diversify the collaboration networks of countries like India and Brazil, enhancing their global research visibility and impact. At the institutional level, the concentration of high-output institutions in France (e.g., Institut Pasteur, Université Paris Cité) and Brazil (e.g., Fundação Oswaldo Cruz) highlights the importance of institutional alliance-building. The close collaborations between French and Brazilian institutions with partners in Europe, North America, and Asia demonstrate the effectiveness of transcontinental research consortia. Future initiatives could formalize these partnerships into structured international research networks focused on specific themes—such as vector biology, vaccine development, or genomic surveillance—enabling shared data platforms, joint funding proposals, and coordinated multi-center trials. Author-level analysis reveals that prolific researchers like Scott C. Weaver often act as collaboration nuclei, bridging multiple institutions and disciplines. Encouraging the formation of theme-based research clusters around such key authors can foster interdisciplinary innovation. For example, integrating virologists, immunologists, epidemiologists, and data scientists within a single collaborative framework could accelerate the translation of basic findings into public health applications. Journal analysis indicates that high-impact journals such as PLOS NEGLECTED TROPICAL DISEASES and VIRUSES BASEL serve as important dissemination channels for CHIKV research. These journals could play a more active role in curating special issues or thematic series that highlight collaborative international studies, thereby incentivizing cross border research partnerships and promoting the integration of diverse datasets and methodologies. To fully unlock the potential of global cooperation, this study proposes exploring the establishment of a CHIKV Research Coordination Platform—a combined digital and organizational infrastructure designed to support real-time data sharing, joint training programs, and coordinated response mechanisms during outbreaks. Such a platform could integrate genomic, epidemiological, and clinical data from multiple countries, thereby facilitating the development of predictive models and equitable vaccine distribution strategies.

According to the cluster analysis in Figures 6, 7, recent research hotspots related to CHIKV have primarily focused on phylogenetic analysis and epidemiology, pathogenesis and drug therapy, as well as vaccine development. Research in phylogenetic analysis and epidemiology includes the following representative studies. Krambrich J et al. investigated the evolution and molecular history of the East/Central/South African (ECSA) genotype to determine the origin of the 2018–2019 CHIKV outbreak in Thailand. Through comparative phylogenetic analysis based on complete CHIKV genomes and protein modeling, the team identified specific mutations in the E1/E2 spike complex E1 K211E and E2 V264A which may alter the virus’s vector competence, transmission efficiency, and pathogenicity (Krambrich et al., 2024). Phadungsombat et al. (2020) found that although the CHIKV strains circulating in Bangladesh and Thailand from 2017 to 2019 both belonged to the Indian Ocean Lineage (IOL) of the East/Central/South African (ECSA) genotype, they lacked the E1-226 mutation and instead acquired novel mutations at positions E1-211 and E2-264. These changes gave rise to a distinct new sub-lineage, which exhibited enhanced transmissibility by *Aedes albopictus* (Phadungsombat et al., 2020). Fabbri et al. (2024), through a combination of active surveillance, screening of historical and recent samples, and whole genome sequencing, identified the Asian lineage from the 2016 outbreak and the ECSA lineage from 2023 in Argentina, reflecting the dominance of specific variants in Latin America. A phylogenetic analysis of 205 confirmed CHIKV infection cases during the 2007 outbreak in Italy showed a high similarity between the strains identified in Italy and those from earlier outbreaks in Indian Ocean islands (Rezza et al., 2007). Khongwichit et al. (2021) systematically described the molecular virology, clinical manifestations, diagnosis, prevalence, genotype distribution, evolutionary relationships, and epidemiology of CHIKV infections in Asian countries over the past 65 years. This comprehensive review provides critical insights for guiding epidemiological studies, improving control and prevention strategies for future CHIKV outbreaks, and supporting the development of novel vaccines and antiviral drugs against CHIKV (Khongwichit et al., 2021).

Research on the pathogenesis of CHIKV, Structural analysis of the crystal structure of nsP2 N-terminal RNA helicase domain (nsP2h) in complex with genomic RNA demonstrated that it engages in stacking interactions with RNA bases via three critical hydrophobic residues (Y161, F164, and F287). The mutation of residue F287 impairs both ATPase activity and RNA-binding capacity, double mutation at Y161 and F164 completely abolishes viral replication (Law et al., 2019). Both domains and the hinge region of MXRA8—the molecular receptor for arthritogenic alphaviruses—interact with residues of the CHIKV envelope protein E1-E2. The stalk region of MXRA8 was found to be critical for CHIKV viral entry (Song et al., 2019). Additionally, a single FGDF motif in the CHIKV nsP3 is sufficient to support viral infection and dissemination in mosquitoes, while a double motif is essential for efficient transmission from mosquito saliva to vertebrate hosts. These findings elucidate the molecular mechanisms underlying the alphavirus transmission cycle and provide new insights for the development of intervention strategies against arboviral diseases (Goertz et al., 2018). The CHIKV nsP1 forms a crown shaped ring structure through the assembly of 12 copies at 2.38 Å resolution, enabling material exchange between the viral replication complex and host cells (Zhang et al., 2021).

The development of antiviral drugs and vaccines is crucial for controlling CHIKV infection and preventing outbreaks. The four and a half LIM domain protein 1 (FHL1) has been identified as a key host factor facilitating CHIKV infection, and the interaction between nsP3 and FHL1 represents a promising target for developing anti-CHIKV therapies (Meertens et al., 2019). Levi et al. (2021) combined experimental and computational approaches to select defective viral genomes (DVGs) with the highest potential inhibitory activity, which function by interfering with CHIKV replication in both mammalian and mosquito cells. They also demonstrated that pre-treating *Aedes aegypti* mosquitoes with DVGs can prevent the transmission of CHIKV strains and other alphaviruses *in vivo* (Levi et al., 2021). Martins et al. (2020) reported that natural compounds such as chloroquine, apigenin, chrysin, flavaglines, and trigocherrierin A exhibit antiviral activity by blocking early and/or late stages of CHIKV replication *in vitro*. The novel compounds thieno[3,2-b] pyrrole 20 and pyrrolo[2,3-d] thiazole 23c demonstrated a 17-fold extension in metabolic half life along with favorable *in vivo* pharmacokinetic properties. Notably, compound 20 effectively suppressed viral RNA replication and exhibited broad spectrum antiviral activity against multiple alphavirus species and CHIKV strains, while maintaining low cytotoxicity (Ching et al., 2017). The application of Mxra8 Fc protein or anti-Mxra8 blocking antibodies reduced chikungunya and O’nyong nyong virus infections, as well as associated foot swelling. Pharmacological interventions targeting Mxra8 offer a novel therapeutic strategy for mitigating infections caused by various arthritogenic alphaviruses and related diseases (Zhang et al., 2018). A 20 μg dose of virus-like particle (VLP) based CHIKV vaccine was shown to be safe and well-tolerated (Jaiswal et al., 2020). A single dose of a chimeric Eilat/Chikungunya virus (EILV/CHIKV) vaccine elicited high titer CHIKV specific antibodies, memory B cells, and CD8^+^ T cell responses, and induced stronger CD8^+^ T cell immunity compared to the live attenuated vaccine strain 181/25. This vaccine candidate activates antigen presenting cells (APCs) and triggers antiviral cytokine responses *in vivo*, but does not induce APC activation *in vitro* alone, offering a favorable safety profile and cost effective production advantages (Adam et al., 2021). Ixchiq is developed using live attenuated vaccine technology and features a large 60 amino acid deletion in the nSP3 protein, resulting in reduced replication efficiency compared to wild-type CHIKV. A single 0.5 mL dose induces high levels of CHIKV neutralizing antibodies immediately after vaccination (Ng and Renia, 2024).

CHIKV research in high output countries exhibits distinct research priorities and collaboration patterns, as revealed by an analysis of the 20 keywords with the strongest citation bursts. Four high impact studies independently published by France covered topics in CHIKV epidemiology and phylogenetic analysis (Leparc-Goffart et al., 2014; Grandadam et al., 2011; Thiberville et al., 2013) and CHIKV complications (Schilte et al., 2013). Two articles from the U.S. focused on CHIKV epidemiology, replication cycle, pathogenesis, and host immune response (Silva and Dermody, 2017), revealing that Mxra8 serves as a receptor binding to domains A and B of the chikungunya virus E2 protein (Zhang et al., 2018). A contribution from Sweden analyzed the historical trends and transmission patterns of chikungunya fever in Africa and Asia (Zeller et al., 2016). Pakistan published a systematic review tracing the 64-year global history of CHIKV infection (Wahid et al., 2017). Australia’s Suhrbier A. reported on CHIKV immunopathology and interventions for CHIKV induced rheumatic diseases (Suhrbier, 2019). A Brazilian article emphasized advances in understanding CHIKV pathogenesis and provided critical insights into the current development and clinical trials of potential CHIKV vaccine candidates (de Lima Cavalcanti et al., 2022). International collaborative papers accounted for 50% (10/20) of these high-impact studies. Collaborative research primarily addressed CHIKV clinical features and complications (Burt et al., 2012), epidemiology and disease burden (Bettis et al., 2022; Puntasecca et al., 2021; Bhatt et al., 2013; Suhrbier et al., 2012), global vector distribution and transmission trends (Kraemer et al., 2015; Kraemer et al., 2019), and pathogenesis (Burt et al., 2017). Notably, a multinational collaboration involving Norway, the UK, the U.S., Belgium, Singapore, and Brazil conducted a statistical analysis of the global epidemiology of chikungunya fever from 1999 to 2020. The study revealed that outbreaks are sporadic and unpredictable, highlighted significant gaps in existing epidemiological data (such as age-specific infection rates), and noted a lack of standardization in research methodologies, all of which pose major challenges to vaccine efficacy trials. Improving dynamic disease surveillance and studying long term population immunity are crucial for vaccine development and post marketing evaluation (Bettis et al., 2022). The strength of international collaboration lies in its ability to integrate expertise from various countries, forming interdisciplinary and cross regional research networks capable of tackling large-scale challenges that are difficult for any single country to address, such as global epidemiological statistics and analysis of disease transmission trends. Collaborative teams can access and analyze data from multiple continents and countries, providing a more comprehensive reflection of the global CHIKV situation. Their conclusions are thus more representative and authoritative, offering a reliable foundation for global vaccine development and prevention strategies.

The research hotspots and frontiers identified through our bibliometric analysis hold significant clinical and public health relevance for shaping global strategies against CHIKV, albeit with distinct implications for developed and developing countries. The persistent focus on pathogenesis and immunopathology (Suhrbier, 2019; Thiberville et al., 2013; Schilte et al., 2013; Suhrbier et al., 2012) is directly relevant to managing the chronic arthralgia that burdens a substantial proportion of CHIKV patients. Understanding the mechanisms of viral persistence and immune mediated joint damage is the first step toward developing targeted therapies beyond symptomatic relief with analgesics and anti inflammatory drugs. This is crucial for improving long term patient outcomes and reducing disability in both resource rich and resource limited settings. Furthermore, the strong research interest in antiviral activity and drug therapy (Zhang et al., 2018; Burt et al., 2017; Meertens et al., 2019; Levi et al., 2021; Martins et al., 2020; Ching et al., 2017) underscores the urgent, unmet clinical need for a specific antiviral drug. The progression of candidate compounds from *in vitro* studies to clinical trials is therefore a critical translational pathway that could revolutionize acute patient management. The intense focus on vaccine development (Ng and Renia, 2024; de Lima Cavalcanti et al., 2022; Jaiswal et al., 2020; Adam et al., 2021; Bettis et al., 2022), reflected in keywords and burst references, addresses the primary preventive need. Our analysis of collaboration patterns and global research output reveals the next challenges: ensuring equitable vaccine access and generating robust real-world efficacy and safety data across diverse genetic and immunological backgrounds, particularly in endemic low and middle income countries (LMICs). Keywords like ‘safety’ and ‘immunogenicity’ (Burt et al., 2017; Weber et al., 2024; Silva and Dermody, 2017; Jaiswal et al., 2020; Adam et al., 2021) emerging as recent hotspots highlight the research community’s awareness of these post licensure considerations. The prospects for CHIKV control are shaped by the different realities of developed and developing nations. In developing countries, particularly in tropical regions of Asia, Africa, and the Americas where CHIKV is endemic, the public health priority remains cost effective surveillance, vector control, and outbreak management. The research hotspot of ‘epidemiology’ and ‘coinfection’ (e.g., with dengue) is highly relevant here, as it informs surveillance systems and differential diagnosis. The development of low cost, rapid diagnostic tests is a direct clinical application of research on viral antigens and antibodies. For these regions, the future challenge lies in integrating new tools like vaccines into existing public health frameworks. The deep but narrow collaboration patterns of countries like Brazil and India, as identified in our analysis, could be leveraged to form regional research consortia focused on context specific vaccine implementation studies and monitoring for any vaccine driven evolutionary changes in the virus.

Developed countries, while facing less endemic transmission, are highly vulnerable to travel-imported cases and localized outbreaks driven by established *Aedes albopictus* populations, as seen in Italy and France. For these nations, the research frontier of transmission risk prediction—integrating climate data, vector maps, and human mobility—is of immediate clinical relevance. It enables targeted public health messaging and pre-emptive vector control. Their research strengths, often in basic virology and immunology (as seen in the output from the U.S. and France), are essential for elucidating pathogenic mechanisms that can inform the development of next generation pan-alphavirus vaccines and therapeutics. The broad and intensive international collaboration networks of the U.S. and European countries should be strategically used to support genomic surveillance capacity in LMICs, creating a global early warning system for emerging variants.

Based on a bibliometric analysis of the CHIKV literature, this study systematically outlines the evolution of CHIKV research and provides an in-depth interpretation of its developmental trajectory and current research landscape. Future studies on CHIKV are likely to focus on the following cutting-edge directions: (1) Transmission Risk Prediction and Global Surveillance: Integrating multi source data such as climate variables, mosquito vector distribution, and human mobility to develop predictive models for dynamic global assessment of CHIKV transmission risk. Simultaneously, establishing a global phylogenomic surveillance network to track viral evolution and dissemination routes in real time. (2) Viral Evolution and Host Adaptation Mechanisms: Investigating key genomic determinants that drive host adaptation mutations in CHIKV, and evaluating their impact on viral transmissibility, virulence, and potential for cross species transmission. (3) Host Pathogen Interactions and Immunopathological Mechanisms: Elucidating the immunological pathways underlying CHIKV induced chronic arthritis, and developing targeted therapeutic strategies to modulate host immunopathological responses. (4) Precision Antiviral Drug Development: Designing specific inhibitors targeting viral structural proteins (e.g., E1/E2) and non structural proteins (e.g., nsP1, nsP2, nsP3), and exploring broad spectrum antiviral strategies effective against CHIKV. (5) Novel Vaccine Development: Accelerating the development of multi platform preventive vaccines, including mRNA based vaccines, live attenuated vaccines, and subunit vaccines, with emphasis on cross protective efficacy and long term immunogenicity. These research directions aim to overcome current bottlenecks in both basic research and clinical applications, thereby providing robust scientific support for global public health responses.

Several limitations should be acknowledged when employing bibliometric methods to systematically analyze the literature related to CHIKV. First, although WoSCC is an authoritative and comprehensive database, it predominantly indexes English-language journals. Consequently, this may lead to the omission of crucial literature, particularly from high incidence regions and top producing countries of CHIKV research, such as Brazil and India. Key studies published in regional journals or in local languages (e.g., Portuguese, Spanish), which may contain vital epidemiological and clinical data on CHIKV, are likely excluded. Second, in the analysis of highly cited literature, we identified an issue of thematic focus dilution. The list of most globally cited articles is predominantly comprised of studies on other arboviruses such as Zika and Dengue. Although these topics are interrelated, the fact that the most influential articles in the “CHIKV field” are not strictly focused on CHIKV itself indicates that citation-based analysis partially reflects the research emphasis driven by concurrent major outbreaks (such as the 2016 Zika epidemic) and shared vector related challenges, rather than capturing the core impact of CHIKV specific fundamental research. Third, the data collection period of this study coincided with the COVID-19 pandemic, during which research focus in virology largely shifted to SARS-CoV-2. The explosive growth of publications related to COVID-19 may have significantly influenced the bibliometric analysis of CHIKV research, potentially obscuring specific research trends and dynamics of CHIKV, thereby introducing unavoidable bias. Furthermore, bibliometric methods are inherently constrained by the time dependent nature of citation accumulation. Since the dissemination and recognition of academic influence require time, recently published articles generally receive far fewer citations than older publications. This may lead to an underestimation of emerging directions and cutting edge topics in CHIKV research.

## 5 Conclusion

Current research on CHIKV primarily focuses on several core areas, including epidemiological surveillance and transmission dynamics, viral pathogenesis and host immune responses, as well as antiviral drug screening and vaccine development. International collaboration has played a crucial role in advancing the understanding of CHIKV transmission mechanisms, elucidating viral evolutionary patterns, and achieving breakthroughs in prevention and control technologies. Future studies should prioritize the following directions: transmission risk prediction and global surveillance, viral evolution and host adaptation mechanisms, host pathogen interactions and immunopathological mechanisms. Precision antiviral drug development and novel vaccine platforms. These efforts aim to overcome current technical bottlenecks in both basic research and clinical applications of CHIKV, further refine the theoretical framework and practical strategies for viral control, provide scientific support for global public health responses to CHIKV threats, and help mitigate its impact on human health and public security.

## Data Availability

The original contributions presented in the study are included in the article/supplementary material, further inquiries can be directed to the corresponding author.

## References

[ref1] Adam A. Luo H. Osman S. R. Wang B. Roundy C. M. Auguste A. J. . (2021). Optimized production and immunogenicity of an insect virus-based chikungunya virus candidate vaccine in cell culture and animal models. Emerg Microbes Infect. 10, 305–316. doi: 10.1080/22221751.2021.1886598, 33539255 PMC7919884

[ref2] Basavappa M. G. Ferretti M. Dittmar M. Stoute J. Sullivan M. C. Whig K. . (2022). The lncRNA ALPHA specifically targets chikungunya virus to control infection. Mol. Cell 82, 3729–3744.e10. doi: 10.1016/j.molcel.2022.08.030, 36167073 PMC10464526

[ref3] Bettis A. A. L'Azou Jackson M. Yoon I. K. Breugelmans J. G. Goios A. Gubler D. J. . (2022). The global epidemiology of chikungunya from 1999 to 2020: a systematic literature review to inform the development and introduction of vaccines. PLoS Negl. Trop. Dis. 16:e0010069. doi: 10.1371/journal.pntd.0010069, 35020717 PMC8789145

[ref4] Bhatt S. Gething P. W. Brady O. J. Messina J. P. Farlow A. W. Moyes C. L. . (2013). The global distribution and burden of dengue. Nature 496, 504–507. doi: 10.1038/nature12060, 23563266 PMC3651993

[ref5] Bierbrier R. Javelle E. Norman F. F. Chen L. H. Bottieau E. Schwartz E. . (2024). Chikungunya infection in returned travellers: results from the geosentinel network, 2005-2020. J. Travel Med. 31. doi: 10.1093/jtm/taae005, 38195993 PMC11081466

[ref6] Brasil P. Pereira J. P. Jr. Moreira M. E. Ribeiro Nogueira R. M. Damasceno L. Wakimoto M. . (2016). Zika virus infection in pregnant women in Rio de Janeiro. N. Engl. J. Med. 15, 2321–2334. doi: 10.1056/NEJMoa1602412, 26943629 PMC5323261

[ref7] Broeckel R. Fox J. M. Haese N. Kreklywich C. N. Sukulpovi-Petty S. Legasse A. . (2017). Therapeutic administration of a recombinant human monoclonal antibody reduces the severity of chikungunya virus disease in rhesus macaques. PLoS Negl. Trop. Dis. 11:e0005637. doi: 10.1371/journal.pntd.0005637, 28628616 PMC5491320

[ref8] Burt F. J. Chen W. Miner J. J. Lenschow D. J. Merits A. Schnettler E. . (2017). Chikungunya virus: an update on the biology and pathogenesis of this emerging pathogen. Lancet Infect. Dis. 17, e107–e117. doi: 10.1016/S1473-3099(16)30385-1, 28159534

[ref9] Burt F. J. Rolph M. S. Rulli N. E. Mahalingam S. Heise M. T. (2012). Chikungunya: a re-emerging virus. Lancet 379, 662–671. doi: 10.1016/S0140-6736(11)60281-X, 22100854

[ref10] Cabie A. Ledrans M. Abel S. (2015). Chikungunya virus infections. N. Engl. J. Med. 373, 93–95. doi: 10.1056/NEJMc1505501, 26132958

[ref11] Cai L. Hu X. Liu S. Wang L. Lu H. Tu H. . (2022). The research progress of chikungunya fever. Front. Public Health 10:1095549. doi: 10.3389/fpubh.2022.1095549, 36699921 PMC9870324

[ref12] Calvet G. Aguiar R. S. Melo A. S. O. Sampaio S. A. de Filippis I. Fabri A. . (2016). Detection and sequencing of Zika virus from amniotic fluid of fetuses with microcephaly in Brazil: a case study. Lancet Infect. Dis. 16, 653–660. doi: 10.1016/S1473-3099(16)00095-5, 26897108

[ref13] Chen R. Plante J. A. Plante K. S. Yun R. Shinde D. Liu J. . (2021). Lineage divergence and vector-specific adaptation have driven chikungunya virus onto multiple adaptive landscapes. MBio 12:e0273821. doi: 10.1128/mBio.02738-21, 34749526 PMC8576524

[ref14] Ching K. C. Tran T. N. Amrun S. N. Kam Y. W. Ng L. F. Chai C. L. (2017). Structural optimizations of Thieno[3,2-b]pyrrole derivatives for the development of metabolically stable inhibitors of chikungunya virus. J. Med. Chem. 60, 3165–3186. doi: 10.1021/acs.jmedchem.7b00180, 28350454

[ref15] Chouin-Carneiro T. Vega-Rua A. Vazeille M. Yebakima A. Girod R. Goindin D. . (2016). Differential susceptibilities of *Aedes aegypti* and *Aedes albopictus* from the Americas to Zika virus. PLoS Negl. Trop. Dis. 10:e0004543. doi: 10.1371/journal.pntd.0004543, 26938868 PMC4777396

[ref16] de Lima Cavalcanti T. Y. V. Pereira M. R. de Paula S. O. Franca R. F. O. (2022). A review on chikungunya virus epidemiology, pathogenesis and current vaccine development. Viruses 14:969. doi: 10.3390/v14050969 35632709 PMC9147731

[ref17] de Souza W. M. Fumagalli M. J. de Lima S. T. S. Parise P. L. Carvalho D. C. M. Hernandez C. . (2024a). Pathophysiology of chikungunya virus infection associated with fatal outcomes. Cell Host Microbe 10, 606–622 e8. doi: 10.1016/j.chom.2024.02.011, 38479396 PMC11018361

[ref18] de Souza W. M. Gaye A. Ndiaye E. H. Morgan A. L. Sylla E. H. D. Sy F. A. . (2024b). Serosurvey of chikungunya virus in Old World fruit bats, Senegal, 2020-2022. Emerg. Infect. Dis. 30, 1490–1492. doi: 10.3201/eid3007.240055, 38916865 PMC11210635

[ref19] Dejnirattisai W. Supasa P. Wongwiwat W. Rouvinski A. Barba-Spaeth G. Duangchinda T. . (2016). Dengue virus sero-cross-reactivity drives antibody-dependent enhancement of infection with zika virus. Nat. Immunol. 17, 1102–1108. doi: 10.1038/ni.3515, 27339099 PMC4994874

[ref20] Erasmus J. H. Auguste A. J. Kaelber J. T. Luo H. Rossi S. L. Fenton K. . (2017). A chikungunya fever vaccine utilizing an insect-specific virus platform. Nat. Med. 23, 192–199. doi: 10.1038/nm.4253, 27991917 PMC5296253

[ref21] Erasmus J. H. Rossi S. L. Weaver S. C. (2016). Development of vaccines for chikungunya fever. J. Infect. Dis. 214, S488–S496. doi: 10.1093/infdis/jiw271, 27920179 PMC5137239

[ref22] Fabbri C. Giovanetti M. Luppo V. Fonseca V. Garcia J. Barulli C. . (2024). Tracing the evolution of the chikungunya virus in Argentina, 2016-2023: independent introductions and prominence of Latin American lineages. Emerg. Microbes Infect. 13:2362941. doi: 10.1080/22221751.2024.2362941, 38813649 PMC11168220

[ref23] Fox J. M. Roy V. Gunn B. M. Huang L. Edeling M. A. Mack M. . (2019). Optimal therapeutic activity of monoclonal antibodies against chikungunya virus requires fc-FcγR interaction on monocytes. Sci. Immunol. 4. doi: 10.1126/sciimmunol.aav5062, 30796092 PMC6698136

[ref24] Freppel W. Silva L. A. Stapleford K. A. Herrero L. J. (2024). Pathogenicity and virulence of chikungunya virus. Virulence 15:2396484. doi: 10.1080/21505594.2024.2396484, 39193780 PMC11370967

[ref25] Goertz G. P. Lingemann M. Geertsema C. Abma-Henkens M. H. C. Vogels C. B. F. Koenraadt C. J. M. . (2018). Conserved motifs in the hypervariable domain of chikungunya virus nsP3 required for transmission by *Aedes aegypti* mosquitoes. PLoS Negl. Trop. Dis. 12:e0006958. doi: 10.1371/journal.pntd.0006958, 30412583 PMC6249005

[ref26] Grandadam M. Caro V. Plumet S. Thiberge J. M. Souares Y. Failloux A. B. . (2011). Chikungunya virus, southeastern France. Emerg. Infect. Dis. 17, 910–913. doi: 10.3201/eid1705.101873, 21529410 PMC3321794

[ref27] Guzman M. G. Gubler D. J. Izquierdo A. Martinez E. Halstead S. B. (2016). Dengue infection. Nat. Rev. Dis. Primers 2:16055. doi: 10.1038/nrdp.2016.55, 27534439

[ref28] Hoornweg T. E. van Duijl-Richter M. K. S. Ayala Nunez N. V. Albulescu I. C. van Hemert M. J. Smit J. M. (2016). Dynamics of chikungunya virus cell entry unraveled by single-virus tracking in living cells. J. Virol. 90, 4745–4756. doi: 10.1128/JVI.03184-15, 26912616 PMC4836339

[ref29] Jaiswal N. Singh S. Singh M. (2020). Chikungunya virus-like particle vaccine. JAMA 324:1008. doi: 10.1001/jama.2020.11845, 32897341

[ref30] Jin J. Galaz-Montoya J. G. Sherman M. B. Sun S. Y. Goldsmith C. S. O'Toole E. T. . (2018). Neutralizing antibodies inhibit chikungunya virus budding at the plasma membrane. Cell Host Microbe 24, 417–428.e5. doi: 10.1016/j.chom.2018.07.018, 30146390 PMC6137268

[ref31] Khongwichit S. Chansaenroj J. Chirathaworn C. Poovorawan Y. (2021). Chikungunya virus infection: molecular biology, clinical characteristics, and epidemiology in Asian countries. J. Biomed. Sci. 28:84. doi: 10.1186/s12929-021-00778-8, 34857000 PMC8638460

[ref32] Kraemer M. U. G. Reiner R. C. Jr. Brady O. J. Messina J. P. Gilbert M. Pigott D. M. . (2019). Past and future spread of the arbovirus vectors *Aedes aegypti* and *Aedes albopictus*. Nat. Microbiol. 4, 854–863. doi: 10.1038/s41564-019-0376-y, 30833735 PMC6522366

[ref33] Kraemer M. U. Sinka M. E. Duda K. A. Mylne A. Q. Shearer F. M. Barker C. M. . (2015). The global distribution of the arbovirus vectors *Aedes aegypti* and *ae. Albopictus*. eLife 4:e08347. doi: 10.7554/eLife.08347, 26126267 PMC4493616

[ref34] Krambrich J. Mihalic F. Gaunt M. W. Bohlin J. Hesson J. C. Lundkvist A. . (2024). The evolutionary and molecular history of a chikungunya virus outbreak lineage. PLoS Negl. Trop. Dis. 18:e0012349. doi: 10.1371/journal.pntd.0012349, 39058744 PMC11305590

[ref35] Kril V. Hons M. Amadori C. Zimberger C. Couture L. Bouery Y. . (2024). Alphavirus nsP3 organizes into tubular scaffolds essential for infection and the cytoplasmic granule architecture. Nat. Commun. 15:8106. doi: 10.1038/s41467-024-51952-z, 39285216 PMC11405681

[ref36] Langsjoen R. M. Auguste A. J. Rossi S. L. Roundy C. M. Penate H. N. Kastis M. . (2017). Host oxidative folding pathways offer novel anti-chikungunya virus drug targets with broad spectrum potential. Antivir. Res. 143, 246–251. doi: 10.1016/j.antiviral.2017.04.014, 28461071

[ref37] Langsjoen R. M. Rubinstein R. J. Kautz T. F. Auguste A. J. Erasmus J. H. Kiaty-Figueroa L. . (2016). Molecular virologic and clinical characteristics of a chikungunya fever outbreak in La Romana, Dominican Republic, 2014. PLoS Negl. Trop. Dis. 10:e0005189. doi: 10.1371/journal.pntd.0005189, 28030537 PMC5193339

[ref38] Law Y. S. Utt A. Tan Y. B. Zheng J. Wang S. Chen M. W. . (2019). Structural insights into RNA recognition by the chikungunya virus nsP2 helicase. Proc. Natl. Acad. Sci. USA 116, 9558–9567. doi: 10.1073/pnas.1900656116, 31000599 PMC6511008

[ref39] Leparc-Goffart I. Nougairede A. Cassadou S. Prat C. de Lamballerie X. (2014). Chikungunya in the Americas. Lancet 383:514. doi: 10.1016/S0140-6736(14)60185-9, 24506907

[ref40] Levi L. I. Rezelj V. V. Henrion-Lacritick A. Erazo D. Boussier J. Vallet T. . (2021). Defective viral genomes from chikungunya virus are broad-spectrum antivirals and prevent virus dissemination in mosquitoes. PLoS Pathog. 17:e1009110. doi: 10.1371/journal.ppat.1009110, 33556143 PMC7870000

[ref41] Lumsden W. H. (1955). An epidemic of virus disease in Southern Province, Tanganyika territory, in 1952-53. II. General description and epidemiology. Trans. R. Soc. Trop. Med. Hyg. 49, 33–57. doi: 10.1016/0035-9203(55)90081-x, 14373835

[ref42] Martins D. O. S. Santos I. A. de Oliveira D. M. Grosche V. R. Jardim A. C. G. (2020). Antivirals against chikungunya virus: is the solution in nature? Viruses 12. doi: 10.3390/v12030272, 32121393 PMC7150839

[ref43] Maure C. Khazhidinov K. Kang H. Auzenbergs M. Moyersoen P. Abbas K. . (2024). Chikungunya vaccine development, challenges, and pathway toward public health impact. Vaccine 42:126483. doi: 10.1016/j.vaccine.2024.126483, 39467413

[ref44] McAllister N. Liu Y. Silva L. M. Lentscher A. J. Chai W. Wu N. . (2020). Chikungunya virus strains from each genetic clade bind sulfated glycosaminoglycans as attachment factors. J. Virol. 94. doi: 10.1128/JVI.01500-20, 32999033 PMC7925169

[ref45] Meertens L. Hafirassou M. L. Couderc T. Bonnet-Madin L. Kril V. Kummerer B. M. . (2019). FHL1 is a major host factor for chikungunya virus infection. Nature 574, 259–263. doi: 10.1038/s41586-019-1578-4, 31554973

[ref46] Moyes C. L. Vontas J. Martins A. J. Ng L. C. Koou S. Y. Dusfour I. . (2017). Contemporary status of insecticide resistance in the major Aedes vectors of arboviruses infecting humans. PLoS Negl. Trop. Dis. 11:e0005625. doi: 10.1371/journal.pntd.0005625, 28727779 PMC5518996

[ref47] Musso D. Gubler D. J. (2016). Zika virus. Clin. Microbiol. Rev. 29, 487–524. doi: 10.1128/CMR.00072-15, 27029595 PMC4861986

[ref48] Ng L. F. P. Renia L. (2024). Live-attenuated chikungunya virus vaccine. Cell 187, 813–813 e1. doi: 10.1016/j.cell.2024.01.033, 38364787

[ref49] Phadungsombat J. Imad H. Rahman M. Nakayama E. E. Kludkleeb S. Ponam T. . (2020). A novel sub-lineage of chikungunya virus east/central/south African genotype Indian Ocean lineage caused sequential outbreaks in Bangladesh and Thailand. Viruses 12. doi: 10.3390/v12111319, 33213040 PMC7698486

[ref50] Puntasecca C. J. King C. H. LaBeaud A. D. (2021). Measuring the global burden of chikungunya and Zika viruses: a systematic review. PLoS Negl. Trop. Dis. 15:e0009055. doi: 10.1371/journal.pntd.0009055, 33661908 PMC7932082

[ref51] Rezza G. Nicoletti L. Angelini R. Romi R. Finarelli A. C. Panning M. . (2007). Infection with chikungunya virus in Italy: an outbreak in a temperate region. Lancet 370, 1840–1846. doi: 10.1016/S0140-6736(07)61779-6., 18061059

[ref52] Robinson M. C. (1955). An epidemic of virus disease in Southern Province, Tanganyika territory, in 1952-53. I. Clinical features. Trans. R. Soc. Trop. Med. Hyg. 49, 28–32. doi: 10.1016/0035-9203(55)90080-8, 14373834

[ref53] Rossi S. L. Comer J. E. Wang E. Azar S. R. Lawrence W. S. Plante J. A. . (2019). Immunogenicity and efficacy of a measles virus-vectored chikungunya vaccine in nonhuman Primates. J. Infect. Dis. 220, 735–742. doi: 10.1093/infdis/jiz202, 31053842 PMC6667792

[ref54] Schilte C. Staikowsky F. Couderc T. Madec Y. Carpentier F. Kassab S. . (2013). Chikungunya virus-associated long-term arthralgia: a 36-month prospective longitudinal study. PLoS Negl. Trop. Dis. 7:e2137. doi: 10.1371/journal.pntd.0002137, 23556021 PMC3605278

[ref55] Schneider M. Narciso-Abraham M. Hadl S. McMahon R. Toepfer S. Fuchs U. . (2023). Safety and immunogenicity of a single-shot live-attenuated chikungunya vaccine: a double-blind, multicentre, randomised, placebo-controlled, phase 3 trial. Lancet 401, 2138–2147. doi: 10.1016/S0140-6736(23)00641-4, 37321235 PMC10314240

[ref56] Silva L. A. Dermody T. S. (2017). Chikungunya virus: epidemiology, replication, disease mechanisms, and prospective intervention strategies. J. Clin. Invest. 127, 737–749. doi: 10.1172/JCI84417, 28248203 PMC5330729

[ref57] Song H. Zhao Z. Chai Y. Jin X. Li C. Yuan F. . (2019). Molecular basis of arthritogenic alphavirus receptor MXRA8 binding to chikungunya virus envelope protein. Cell 177, 1714–1724 e12. doi: 10.1016/j.cell.2019.04.008, 31080063

[ref58] Strauss J. H. Strauss E. G. (1994). The alphaviruses: gene expression, replication, and evolution. Microbiol. Rev. 58, 491–562. doi: 10.1128/mr.58.3.491-562.1994, 7968923 PMC372977

[ref59] Suhrbier A. (2019). Rheumatic manifestations of chikungunya: emerging concepts and interventions. Nat. Rev. Rheumatol. 15, 597–611. doi: 10.1038/s41584-019-0276-9, 31481759

[ref60] Suhrbier A. Jaffar-Bandjee M. C. Gasque P. (2012). Arthritogenic alphaviruses–an overview. Nat. Rev. Rheumatol. 8, 420–429. doi: 10.1038/nrrheum.2012.64, 22565316

[ref61] Thiberville S. D. Moyen N. Dupuis-Maguiraga L. Nougairede A. Gould E. A. Roques P. . (2013). Chikungunya fever: epidemiology, clinical syndrome, pathogenesis and therapy. Antivir. Res. 99, 345–370. doi: 10.1016/j.antiviral.2013.06.009, 23811281 PMC7114207

[ref62] Tsetsarkin K. A. Chen R. Weaver S. C. (2016). Interspecies transmission and chikungunya virus emergence. Curr. Opin. Virol. 16, 143–150. doi: 10.1016/j.coviro.2016.02.007, 26986235 PMC4824623

[ref63] Tsetsarkin K. A. Vanlandingham D. L. McGee C. E. Higgs S. (2007). A single mutation in chikungunya virus affects vector specificity and epidemic potential. PLoS Pathog. 3:e201. doi: 10.1371/journal.ppat.0030201, 18069894 PMC2134949

[ref64] Wahid B. Ali A. Rafique S. Idrees M. (2017). Global expansion of chikungunya virus: mapping the 64-year history. Int. J. Infect. Dis. 58, 69–76. doi: 10.1016/j.ijid.2017.03.006, 28288924

[ref65] Wang M. Wang L. Leng P. Guo J. Zhou H. (2024). Drugs targeting structural and nonstructural proteins of the chikungunya virus: a review. Int. J. Biol. Macromol. 262:129949. doi: 10.1016/j.ijbiomac.2024.129949, 38311132

[ref66] Weaver S. C. Charlier C. Vasilakis N. Lecuit M. (2018). Zika, chikungunya, and other emerging vector-borne viral diseases. Annu. Rev. Med. 69, 395–408. doi: 10.1146/annurev-med-050715-105122, 28846489 PMC6343128

[ref67] Weaver S. C. Chen R. Diallo M. (2020). Chikungunya virus: role of vectors in emergence from enzootic cycles. Annu. Rev. Entomol. 65, 313–332. doi: 10.1146/annurev-ento-011019-025207, 31594410

[ref68] Weaver S. C. Forrester N. L. (2015). Chikungunya: evolutionary history and recent epidemic spread. Antivir. Res. 120, 32–39. doi: 10.1016/j.antiviral.2015.04.016, 25979669

[ref69] Weaver S. C. Lecuit M. (2015). Chikungunya virus and the global spread of a mosquito-borne disease. N. Engl. J. Med. 372, 1231–1239. doi: 10.1056/NEJMra1406035, 25806915

[ref70] Weber W. C. Streblow D. N. Coffey L. L. (2024). Chikungunya virus vaccines: a review of IXCHIQ and PXVX0317 from pre-clinical evaluation to licensure. BioDrugs 38, 727–742. doi: 10.1007/s40259-024-00677-y, 39292392 PMC11530495

[ref71] Wilder-Smith A. B. Wilder-Smith A. (2024). Determining force of infection for chikungunya to support vaccine policy development. Lancet Infect. Dis. 24, 441–442. doi: 10.1016/S1473-3099(24)00062-8, 38342104

[ref72] Zanluca C. Melo V. C. Mosimann A. L. Santos G. I. Santos C. N. Luz K. (2015). First report of autochthonous transmission of Zika virus in Brazil. Mem. Inst. Oswaldo Cruz 110, 569–572. doi: 10.1590/0074-02760150192, 26061233 PMC4501423

[ref73] Zeller H. Van Bortel W. Sudre B. (2016). Chikungunya: its history in Africa and Asia and its spread to new regions in 2013-2014. J. Infect. Dis. 214, S436–S440. doi: 10.1093/infdis/jiw391, 27920169

[ref74] Zhang R. Kim A. S. Fox J. M. Nair S. Basore K. Klimstra W. B. . (2018). Mxra8 is a receptor for multiple arthritogenic alphaviruses. Nature 557, 570–574. doi: 10.1038/s41586-018-0121-3, 29769725 PMC5970976

[ref75] Zhang K. Law Y. S. Law M. C. Y. Tan Y. B. Wirawan M. Luo D. (2021). Structural insights into viral RNA capping and plasma membrane targeting by chikungunya virus nonstructural protein 1. Cell Host Microbe 29, 757–764.e3. doi: 10.1016/j.chom.2021.02.018, 33730549

